# Is Lateral Elbow Tendinopathy an Appropriate Clinical Diagnostic Term When the Condition Is Persistent?

**DOI:** 10.3390/jcm11092290

**Published:** 2022-04-20

**Authors:** Dimitrios Stasinopoulos, Marianna Papadopoulou

**Affiliations:** 1 Department of Physiotherapy, Faculty of Health and Caring Sciences, University of West Attica, Agiou Spyridonos 28, Egaleo, 12243 Athens, Greece; 2 Department of Neurology, Faculty of Health and Caring Sciences, University of West Attica, Agiou Spyridonos 28, Egaleo, 12243 Athens, Greece; mpapad@uniwa.gr

Lateral elbow tendinopathy (LET) seems to be the most appropriate term to use in clinical practice because previous terms such as lateral epicondylitis, tennis elbow, lateral epicondylalgia, epicondylosis, enthesopathy, Father of the Bride’s Elbow, lateral elbow or extensor tendonitis, lateral elbow or extensor tendinosis, and extensor tendinopathy make reference to inappropriate aetiological, anatomical, and pathophysiological terms [1].

The question that arises is whether the term LET is appropriate when there is persistent LET (PLET). PLET needs to be no longer acknowledged solely as tendinopathy, and instead begin to be understood in part as a disorder with the involvement of the biochemical milieu at the Extensor Digitalis Communis and the radial nerve [2]. In addition, reviewed observational studies provide initial evidence for the assumption that myofascial pain and prevalence of myofascial trigger points co-exists with, causes, or is predisposed to PLET [3]. Finally, patients with PLET present cervical and thoracic spondylosis even though the role of cervical and thoracic spine spondylosis in the prognosis of PLEP requires validation [4,5]. Based on the above, the clinical diagnostic term LET seems to be inappropriate for PLET.

However, the term PLET is not clear in the literature. The term PLET is ranged in the literature from some weeks to many months after the first onset [6]. A patient with 4 weeks of LET probably does not have the same symptoms as a patient with 6 months of LET. The medical society does not define the term PLET. If the medical society defines the term PLET, an appropriate clinical diagnostic term will be recommended, and a gold standard treatment might be found.

The umbrella term lateral elbow pain for PLET has been used in previous Cochrane reviews [7]. However, this term is probably too general for clinical diagnosis, since there are many conditions that can cause pain in the lateral elbow such as LET, radial tunnel syndrome/posterior interosseous neuropathy, distal biceps tendinopathy, osteochondritis dissecans, lateral ulnar collateral ligament instability (posterior lateral elbow rotary instability), posterior lateral elbow plica (lateral synovial fringe), radiocapitellar osteoarthrosis, and cervical radiculopathy [4,5]. Therefore, the umbrella term lateral elbow pain for PLET seems to be inappropriate.

A recommended diagnostic clinical term for PLET might be “lateral elbow pain syndrome” [8]. The meaning of syndrome is a group of signs and symptoms that occur together and characterize a particular abnormality or condition. The term lateral elbow pain syndrome (LEPS) may be a label that references a variety of lateral elbow pain diagnoses such as tendinopathy, nerve involvement, myofascial trigger points, and neck/thoracic dysfunction, all involved in PLET.

An exercise program, supervised or in clinic, is the most effective physiotherapy approach in the management of LET [6]. However, according to previous reported issues, it is time to stop using the exercise program as monotherapy for the treatment of PLET. Neural stretching, myofascial trigger point therapy, and cervical/thoracic mobilization have to be used as adjuncts to exercise programs for the management of PLET. All the other recommended physiotherapy treatments such as electrotherapeutic modalities, manual therapy techniques, soft tissue manipulation, external support, and acupuncture can be used as a supplement to the recommended treatment protocol for the management of PLET [6].

In conclusion, the term lateral elbow pain refers to causes of lateral elbow pain. The term LET is appropriate for clinical diagnosis when this refers to a painful overuse tendon condition. LEPS seems to be appropriate diagnostic clinical term for PLET. A debate on this topic is most welcome, as existing terms may contribute to misunderstanding and inappropriate treatment.
